# The plant AlcR-*pAlcA* ethanol-inducible system displays gross growth artefacts independently of downstream *pAlcA-*regulated inducible constructs

**DOI:** 10.1038/s41598-020-80903-z

**Published:** 2021-01-25

**Authors:** Ricardo S. Randall

**Affiliations:** Department of Plant Developmental Genetics, IPMB, The University of Zürich, Zürich, Switzerland

**Keywords:** Genetic techniques, Genetic engineering, Biological techniques, Developmental biology, Plant sciences

## Abstract

The AlcR fungal protein responds to ethanol and binds to the fungal *pAlcA* promoter in its presence. This system was transferred to plants over twenty years ago and was claimed to function in the same manner in plants. However, never has the control experiment with plants containing the *AlcR* gene alone, with no downstream inducible construct, been made. In this paper, I conduct several experiments with this control, growing *p35:AlcR* plants in the presence or absence of ethanol. I found that when these plants were grown in the presence of ethanol, growth in several tissues and several stages of growth was retarded. This demonstrates that this system is not suitable for use in the plant sciences, and casts doubt on the conclusions of papers that have published phenotypes using this system.

## Introduction

**Figure 1:**
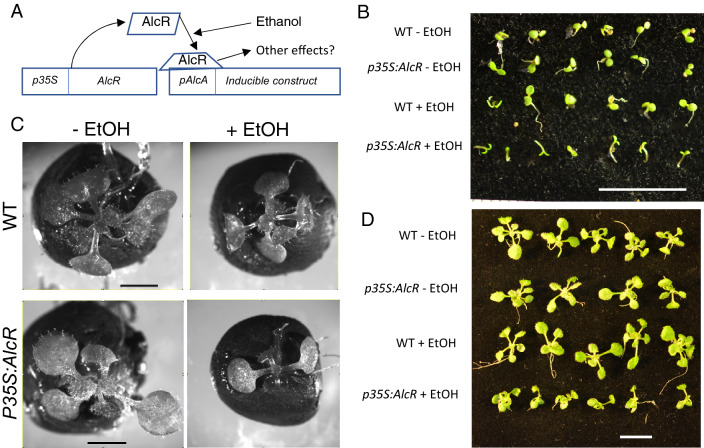
Post germination growth is retarded in *p35S:AlcR* seedlings. (**A**) Model of the supposed mechanism of action of the ethanol-inducible system in plants. (**B**) 3 DAS seedlings grown together in the presence of ethanol since stratification. Reduced growth can be seen in the bottom row. Scale bar = 10 mm. (**C**) Ethanol was added to a subset of seedlings at 3 DAS and images were taken at 7 DAS. Scale bar = 3 mm. (**D**) Ethanol was added at 6 DAS and images taken at 12 DAS. Again, growth is retarded in the *p35S:AlcR* seedlings when grown in the presence of ethanol. Scale bar = 10 mm.

In 1998, Caddick et al.^1^ cloned the *AlcR* gene from *Aspergillus nidulans* along with the *pAlcA* promoter from the same species. Within this fungus, the AlcR protein responds to the presence of ethanol by activating an alcohol dehydrogenase gene, which is regulated by the *pAlcA* promoter that is bound by activated AlcR protein. It was demonstrated^1^ that induction of the expression of genes under the control of the *pAlcA* promoter occurred in plants expressing *AlcR* in the presence of ethanol. It was thus proclaimed that this system could be used for tissue- or developmental-stage induction of genes or other genetic objects using the simple ethanol molecule—as vapour or liquid—which is non-toxic to plants in doses well above those required for induction (Fig. 1A).

However, one fundamental control was missing in this publication^1^: plants lacking the *pAlcA* promoter and any downstream inducible construct were never analysed. It was therefore not shown whether or not the AlcR protein, in the presence of ethanol, had any effect on plant growth and development. This fundamental control has never been included in any publications that I have read that utilised this system. For example, shortly after the publication of the use of this system in plants, tissue-specific induction of various genes in Arabidopsis plants by placing the *AlcR* gene under the control of tissue-specific promoters was shown^2^, but again, no control with plants expressing the *AlcR* gene alone and grown in the presence of ethanol was shown.

ABP1 (Auxin-binding protein 1) was the first protein reported to bind auxin as a receptor in plants. Early studies on an ABP1 orthologue in *Zea mays* showed that this ortholog of Arabidopsis ABP1 preferentially bound to an artificial auxin equivalent^3^. In vitro studies contributed to the hypothesis that ABP1 was a membrane-associated auxin binding protein^4^. Many studies went on to identify phenotypes of *abp1* knock-down mutants, establishing ABP1 as an important auxin receptor required for many growth and developmental processes. Since an initial *abp1* T-DNA insertion line was embryo-lethal, other mutants and means to knock down *ABP1* were sought. An alternative insertion mutant was identified^4^ and was used in combination with knock down mutants in several studies.

In 2015, Gao et al.^5^ used CRISPR-Cas9 technology to create novel *abp1* null mutants. They showed that independently-derived null mutants showed none of the previously described knock-down or insertion mutant phenotypes, and that the plants grew in a manner indistinguishable from WT plants. Furthermore, the plants remained as sensitive to auxin as WT plants. Thence came the question of why all of the previous publications showing *abp1* knock-down phenotypes apparently drew the wrong conclusions. Michalko et al.^6^ showed that embryo-lethality in early T-DNA mutants was due to an additional T-DNA insertion in the *BSM* gene.

Many of the studies that had analyzed *abp1* knock-down phenotypes used the ethanol-inducible system to induce *ABP1-antisense* RNA and antibody peptide fragments that were assumed to immobilise ABP1 and prevent it from functioning. In 2016 it was shown that *abp1* null mutants demonstrated phenotypes only when the antisense RNA or antibody fragments were induced^7^. It was concluded that the phenotypes were due to off-target effects of the antisense RNA and antibody fragments, and not due to knock-down of the *ABP1* gene. However, it was not considered that the ethanol inducible system itself might be causing artefacts.

Here, growth phenotypes of plants expressing the *AlcR* gene under the control of the constitutively active Cauliflower Mosaic virus *p35S* promoter in the absence of any downstream inducible construct are observed.

## Results

### Post-germination growth is retarded in *p35S:AlcR* seedlings in the presence of ethanol vapour

*p35S:AlcR* plants were PCR genotyped to confirm that no *pAlcA* promoter was present (Supp. Fig. 1). Initially, post-germination growth was observed in WT and *p35S:AlcR* seedlings in the presence and absence of 50% ethanol vapour (Fig. 1B). Growth proceeded equally in WT seedlings in the presence and absence of ethanol, whereas post-germination growth was retarded in *p35S:AlcR* seedlings in the presence of ethanol. In the absence of ethanol, *p35S:AlcR* seedlings grew normally. Since a retarded growth phenotype was observed in *p35S:AlcR* seedlings in the presence of ethanol, ethanol was added at later stages of growth in all further experiments.

### Seedling growth is retarded in *p35S:AlcR* seedlings in the presence of ethanol vapour

Tubes containing 50% v/v ethanol were added to *WT* and *p35S:AlcR* seedling-containing plates at 7 DAS (days after stratification). At 12 DAS, WT seedlings in the presence and absence of ethanol, as well as *p35S:AlcR* seedlings, grew at similar rates, showing the growth of true leaves (Fig. 1C). *p35S:AlcR* seedlings grown in the presence of ethanol were retarded in seedling growth, with only the appearance of small juvenile leaves, while other seedlings had already grown several true leaves (Fig. 1C). To confirm these findings, seedlings were grown for 5 days without ethanol, following which 50% ethanol was added. Images of seedlings were then taken after 12 days of growth (7 days in the presence of ethanol) (Supp. Fig. 2). These images confirm that *pAlcR* seedlings display retarded growth in the presence of ethanol.

### Rosette growth is retarded in *p35S:AlcR seedlings* in the presence of ethanol

To observe the effect of the AlcR protein in the presence of ethanol, *WT* and *p35S:AlcR* seedlings were grown in the absence of ethanol for 7 days, after which ethanol was added to *ca*. half of the population of seedlings. Since the 50% v/v ethanol vaporises, ethanol was replenished every two days during further growth. While *WT* rosettes in the presence and absence of ethanol, as well as *p35S:AlcR* rosettes in the absence of ethanol, grew normally, *p35S:AlcR* rosettes in the presence of ethanol demonstrated retarded growth (Fig. 1D).

### Root growth is retarded in *p35S:AlcR* seedlings in the presence of ethanol

**Figure 2:**
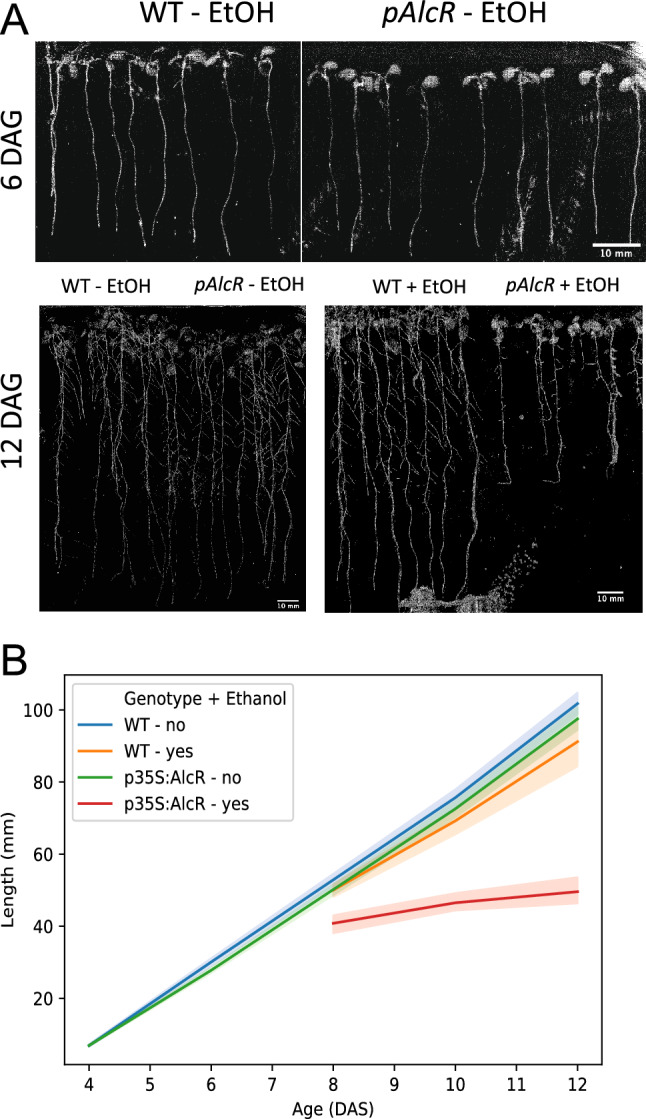
Root growth is retarded in *p35S:AlcR* seedlings in the presence of ethanol, while WT seedling growth proceeds normally (**A**, **B**). Ethanol was added at 6 DAS. (**A**) Scans of roots at the end of the experiment. Scale bar = 10 mm. (**B**) Line plots showing the progression of growth under all conditions. At least ten roots were measured per data point. Shaded areas surrounding lines lines represent 95% confidence intervals.

To analyse the effects of ethanol vapour on *p35S;AlcR* seedling roots, ethanol was added (or not) to 6 DAS WT and *p35S:AlcR* seedlings. Ethanol was again replenished every two days to counteract vaporisation and subsequent loss of ethanol. While root growth proceeded normally in both *WT* and *p35S:AlcR* plants until 6 DAS, growth slowed dramatically in *p35S:AlcR* plants in the presence of ethanol after 6 DAS (Fig. 2A,B).

## Discussion

The results described here demonstrate that the AlcR-*pAlcA* ethanol inducible system used in many plant science projects over the last two decades has a gross growth artefact phenotype. Since the phenotype is only present when ethanol is present, it would appear that the phenotype has nothing to do with the insertion site of the construct but is rather solely due to the presence of activated AlcR protein.

Many of the projects utilizing the system were studies of ABP1 function in *Arabidopsis thaliana*^1, 2, 4, 8^. For example, reduced cell division activity and retarded post embryonic shoot development was described following induction of *ABP1* antisense RNA and ABP1-sequestering antibody-derived peptide fragments^8^. Chen et al.^9^ analysed this cellular growth phenotype at a cellular level, but again employed the ethanol inducible system. They observed inverted microtubule orientation following ethanol induction, the reversed orientation explaining the reduced cell elongation phenotype. As show in Fig. 1, this phenotype could easily be due to the presence of activated AlcR protein. However, the system has been used to study the function of various other genes. For example, Zürcher et al.^10^ used the system to knock down genes involved in the establishment of cytokinin sinks. Phenotypes in root cells were observed; these phenotypes could result from the *p35S:AlcR* phenotype, since root growth was retarded in the presence of activated AlcR (Fig. 2). Since the artefact phenotype seems to be global and strong, it is likely that most if not all of the phenotypes reported in these publications arise from the effects of activated AlcR protein, and thus the conclusions of these publications will most likely be false.

To prevent any further waste of resources, including researchers’ work input, this system should cease to be used in plants. Furthermore, publications that used this system to demonstrate phenotypes^11–18^ should be heavily revised or removed from the literature, since their remaining presence will lead some researchers to plan projects based on false information. Macro phenotypes arise from micro phenotypes, and thus publications investigating micro-cellular effects when the ethanol-inducible system is used to induce some gene or other genetic object should just as importantly be reviewed.

One should also beware of other versions of the ethanol inducible system that have been developed and still utilise the AlcR protein^19–21^.

## Materials and methods

### Plant lines and media

*P35S:AlcR* (N67789) and *p35S:AlcR pAlcA:LHY* (N67791) lines were obtained from NASC, Loughborough, UK. Seeds used, including *WT Col-0*, were obtained from plants grown side-by-side prior to experiments using the harvested seeds. ½ MS media containing Gamborg B5 Vitamins was used for growth. Light intensity for all experiments was ca. 100 µE. Temperature was maintained at 22 °C.

### Ethanol induction

200 mL tubes containing 100 µL 50% v/v were mobilised in plates by partially embedding them in the growth media. Ethanol was replenished every two days following initial addition to counteract evaporation.

### Images of seedlings

Colour seedling images were taken using the *Nikon D7000* DSLR camera. Grey scale images were taken using the *Leica M60 ergo* dissecting microscope.

### Root growth measurements

Root growth was manually measured in Fiji. For plotting, Python Pandas (https://pandas.pydata.org) and Seaborn https://seaborn.pydata.org) packages were used.

## Supplementary Information

Supplementary Figures.
