# Supplementary material for: The plant AlcR-pAlcA ethanol-inducible system displays gross growth artefacts independently of downstream pAlcA-regulated inducible constructs
Source: Sci Rep. 2021 Jan 25;11:2142. doi: 10.1038/s41598-020-80903-z (PMC7835360; doi:10.1038/s41598-020-80903-z)
Supplement: Supplementary file 1 — Supplementary Figures. [file 41598_2020_80903_MOESM1_ESM.pdf]

# The plant AlcR-*pAlcA* ethanol-inducible system displays gross growth artefacts independently of downstream *pAlcA*-regulated inducible constructs

Author:  Ricardo S Randall

## Supplementary Material

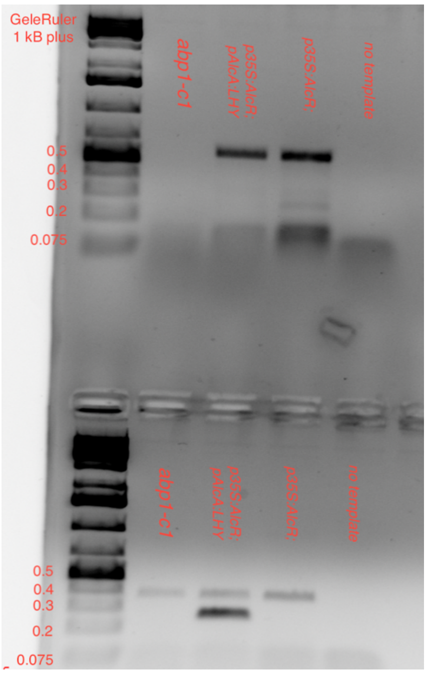


Supp. Fig. 1: PCR genotyping ensures that the *p35S:AlcR* line has no DNA sequence containing the responsive *pAlcA* promoter. Top: primers specific for the *AlcR* gene (5' ATGGCAGATACGCGCCGACG 3'; 5' TCATCCGTGTTCGGGCTATG 3'; expected product size ca. 550 bp) show that the *pAlcR* sequence is present in *p35S:AlcR* plants as well as *p35S:Alcr pAlcA:LHY* control plants. Bottom: primers specific for the *pAlcA* gene (5' CGGGATAGTTCCGACCTAGG 3'; 5' TTATATAGATGTTCAGCTATGCG 3' ; expected product size = 246 bp) show that the *pAlcA* promoter is present in *p35S:AlcR pAlcA:LHY* plants but absent in *p35S:AlcR* control plants.

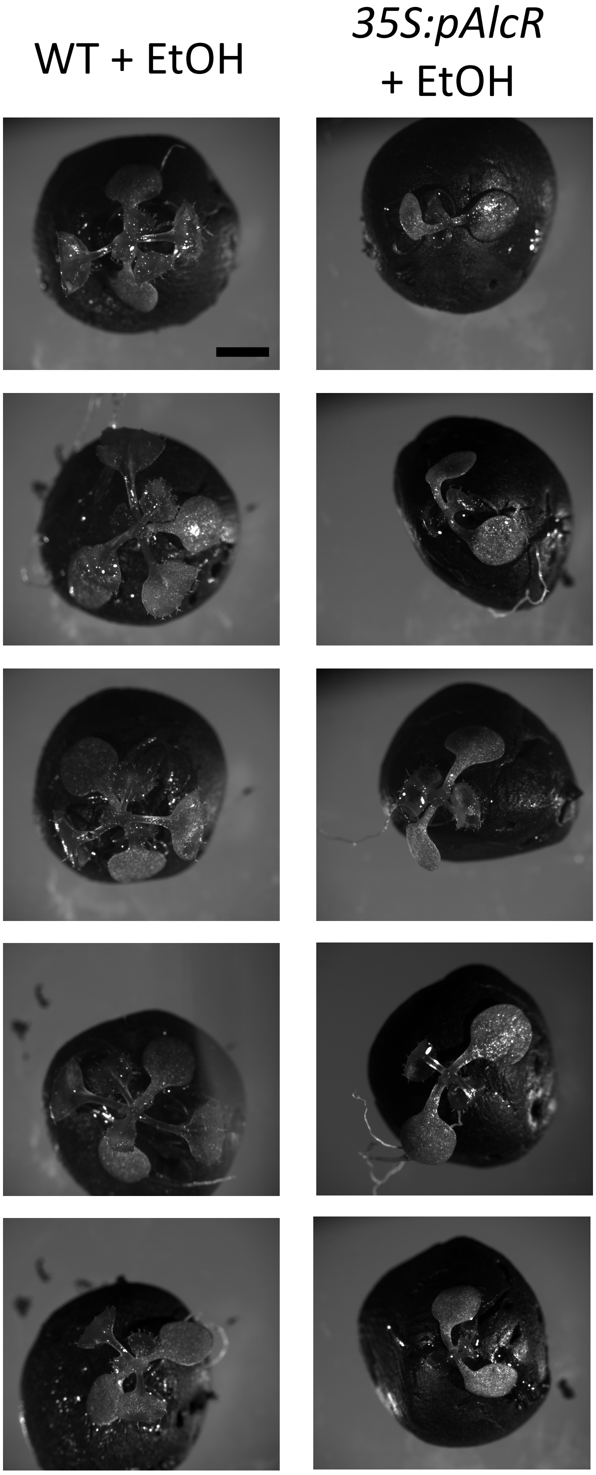


Supp. Fig. 2: 12 DAS WT and *p35S:AlcR* seedlings grown in the presence of 50% v/v ethanol from 5DAS. Scale bar = 3 mm. All images are to scale with respect to one another.
